# Digestive Stasis of the Bowels in the Cow

**Published:** 1900-06

**Authors:** Herbert Hoopes

**Affiliations:** Bynum, Md.


### DIGESTIVE STASIS OF THE BOWELS IN THE COW.

By Herbert Hoopes, V.M.D.,

BYNUM, MD.

I wish to report a recent case of mine and see if my professional brethren can give me any enlightenment on it, as it makes the third case of its kind we have had in our herd, and have not been able to give relief at any time, and as our cows are valuable it is of importance to us as breeders if to no others. One was a registered Jersey cow, just past four years old, in good working order, and had been doing, since calving in June, 1899, a good business at the pail, and was about five months advanced in pregnancy. On Sunday night, February 4th, we had a very heavy rain storm, and during the night the water worked its way through the barn wall, and filled the gutters of one stable, and this cow lay back as far as the stanchion would allow, and then used her tail, and by morning was wet from head to foot, and refused to eat her feed that morning; in fact, I believe had not cleaned up her Sunday night's mess.

I was away all day Monday, and did not learn of her condition until Tuesday morning, when I found her still refusing to eat, occasionally taking a swallow of water; rumination ceased, peristalsis ceased, temperature normal, heart full, strong, and regular, and respiration normal; but the cow seemed to have slight colicky pains, moving from one hind foot to the other and backward and forward.

My diagnosis was gastro-intestinal paralysis. Tuesday morning I gave strychnine sulphate, three-quarters of a grain, hypodermatically, and a drench of one and one-half pounds of magnesium sulphate, with two ounces of pulverized African ginger, and as no result was apparent by 4.30 P.M., gave three grains of pilocarpine and one and three-quarter grains of eserine salicylate, and at 6 P.M. three-quarters grains of strychnine sulphate, and before going to bed gave tr. digitalis, ʒj; tr. nucis vomicæ, ʒss; alcoholis ethylici, ʒj. Milk, q. s., add for drench.

The eserine and pilocarpine produced profused salivation and trembling of the muscles of the abdomen and posterior extremities, and a gurgling sound in the rumen and intestines, but apparently no peristalsis.

Continued twice daily with one grain of strychnine sulphate and once daily with cardiac stimulants, but could see little change until about Friday, when noticed that the heart was losing its force and eyes began to have a sunken appearance.

As a last resort, on Friday afternoon I gave hypodermatically three grains of pilocarpine and two and a quarter grains of eserine, but got no better results than at first.

The cow was able to get up until Sunday night, February 11th, and died Monday morning, February 12th.

Aside from the above treatment I used a funnel and about five feet of rubber hose, and gave injection (on Tuesday and Thursday) of warm water containing stimulants, and worked the tube in four feet and used two and one-half gallons of water at one time. Postmortem revealed that there was no obstruction at any place along the alimentary canal, and that the entire contents of the four stomachs and the intestines was in a semifluid state; the mucus in the lower bowel had become black and partially decomposed, and under the stimulus of the last dose of eserine some of this mucus had been voided.

The cow was apparently healthy in every other respect. Temperature had remained normal until Sunday afternoon, February 11th, when it gradually went below normal.

The other two cases occurred just about one year ago, while I was at school, but from the history they were very similar, except they had no wetting or shock of any kind to bring on the condition, if this was the cause of it.

All three were getting the regular herd feed. If the profession can enlighten me upon this subject the same will be gratefully received.

